# The associations of body shape and composition with incident dementia and cognitive changes

**DOI:** 10.1002/alz.088045

**Published:** 2025-01-09

**Authors:** Zimu Wu, Alice Owen, Robyn Woods, Lachlan Cribb, Zhen Zhou, Trevor T.‐J. Chong, Suzanne Orchard, Raj C. Shah, Rory Wolfe, John J McNeil, Kerry M. Sheets, Anne Murray, Joanne Ryan

**Affiliations:** ^1^ Monash University, Melbourne, VIC Australia; ^2^ Alfred Health, Melbourne, VIC Australia; ^3^ Turner Institute for Brain and Mental Health & School of Psychological Sciences, Monash University, Clayton, VIC Australia; ^4^ St Vincent’s Hospital, Melbourne, VIC Australia; ^5^ Rush University Medical Center, Chicago, IL USA; ^6^ Rush Alzheimer’s Disease Center, Chicago, IL USA; ^7^ Hennepin Healthcare, Minneapolis, MN USA; ^8^ Berman Center for Outcomes and Clinical Research, Hennepin Healthcare Research Institute, Minneapolis, MN USA; ^9^ University of Minnesota, Minneapolis, MN USA

## Abstract

**Background:**

Although the importance of body weight in later life for brain health has been established, less is known about body shape and composition. This study aims to examine their associations with dementia and cognitive changes in older adults.

**Method:**

Data were obtained from over 17,000 community‐dwelling individuals aged 65‐98 years, recruited in Australia and the US. We assessed body shape using the ratio of waist circumference to body mass index (WBR), and estimated lean (LM) and fat mass (FM) by the Hume formula. Dementia diagnosis was adjudicated according to DSM‐IV. Global cognition, verbal fluency, episodic memory, and psychomotor speed were assessed annually for 11 years. Cox regression and mixed‐effects models were used respectively, to estimate the associations with incident dementia and cognitive changes over time.

**Result:**

A higher WBR was associated with higher dementia risk in men (HR: 1.41, 95% CI: 1.04‐1.93, p = 0.03), and with greater decline in all cognitive tests in both genders (coefficients: ‐0.123 to ‐0.030, all p‐values<0.05). Conversely, lower dementia risk was shown in greater LM (men, HR: 0.97, 95% CI: 0.95‐0.99, p<0.001; women, HR: 0.96, 95% CI: 0.95‐0.98, p<0.001) and FM (men, HR: 0.62, 95% CI: 0.48‐0.80, p<0.001; women, HR: 0.63, 95% CI: 0.49‐0.81, p<0.001). Also, they showed slower decline in global cognition, episodic memory, and psychomotor speed (coefficients: 0.002 to 0.007, all p‐values<0.05).

**Conclusion:**

Higher body weight in later life, independent of composition, may benefit brain health. However, increased abdominal fat poses risks for cognitive impairments.

